# *Triatoma guazu* Lent and Wygodzinsky Is a Junior Synonym of *Triatoma williami* Galvão, Souza and Lima

**DOI:** 10.3390/insects13070591

**Published:** 2022-06-28

**Authors:** João Paulo Sales Oliveira Correia, Hélcio Reinaldo Gil-Santana, Carolina Dale, Cleber Galvão

**Affiliations:** 1 Laboratório Nacional e Internacional de Referência em Taxonomia de Triatomíneos, Instituto Oswaldo Cruz, Fiocruz, Av. Brasil 4365, Rio de Janeiro 21040-900, Brazil; joao.correia@ioc.fiocruz.br; 2 Laboratório de Diptera, Instituto Oswaldo Cruz, Fiocruz, Av. Brasil 4365, Rio de Janeiro 21040-360, Brazil; helciogil@ioc.fiocruz.br; 3 Laboratório de Biodiversidade Entomológica, Instituto Oswaldo Cruz, Fiocruz, Av. Brasil 4365, Rio de Janeiro 21040-900, Brazil; dale@ioc.fiocruz.br

**Keywords:** Triatominae, morphological analysis, geometric morphometry, mitochondrial DNA, synonym

## Abstract

**Simple Summary:**

Triatomines are blood-sucking insects, potential vectors of *Trypanosoma* *cruzi*, the etiological agent of Chagas disease. *Triatoma guazu* and *Triatoma williami* are phylogenetically very close and occur in sympatry. Morphologic, morphometric, and genetic analyses were performed to discuss the taxonomic *status* of these species. Morphometric and molecular data do not show diagnostic characteristics between species, whereas their different patterns of connexival spots were considered a phenotypic polymorphism, common in triatomines. These results suggest *T. guazu* as a junior synonym of *T. williami*. Therefore, the synonym between these species is formally proposed here.

**Abstract:**

*Triatoma guazu* Lent and Wygodzinsky and *Triatoma williami* Galvão, Souza, and Lima (Hemiptera: Triatominae) are found in human dwellings and are potential vectors of the protozoan *Trypanosoma cruzi*, the etiological agent of Chagas disease. *Triatoma guazu* was described based solely on a single female specimen, from the municipality of Villarica, Guairá Department, Paraguay, and posteriorly, a male from Barra do Garças, Mato Grosso, Brazil was described and designated as the allotype of this species. *Triatoma williami* is found in the central-west of Brazil between Goiás, Mato Grosso, and Mato Grosso do Sul. However, the taxonomic “status” of these species is questioned. Previous studies indicate the lack of isoenzymatic diagnostic loci, morphometric similarity, low genetic divergence, and close evolutionary relationship of these species. In this study, we compared the morphology, morphometry, and mitochondrial DNA fragments of the populations of the two species. The morphological diagnostic characteristic among these species is the difference in the connexivum spots pattern, which has been recognized as a phenotypic variation that exists among populations resulting from ecological diversity. Furthermore, our analysis also revealed the morphometric similarity and low genetic divergence between these species. Therefore, in the present paper, we formally propose *T. guazu* as a junior synonym of *T. williami*.

## 1. Introduction

The subfamily Triatominae (Jeannel, 1919) includes 155 extant and three fossil species distributed in 18 genera and five tribes [1,2,3,4]. Among the triatomine genera, *Triatoma* Laporte, 1832 has the highest diversity, with 82 species, of which 39 occur in Brazil [5,6,7,8].

*Triatoma williami* Galvão, Souza, and Lima, 1965 was described based on specimens from the municipality of Piranhas, Goiás state, in honor of Dr. William Barbosa for his efforts to create a research institute in the region (Instituto de Patologia Tropical) [9]. This species also occurs in other states of the Cerrado, the tropical savanna ecoregion located in central-west Brazil, such as Mato Grosso and Mato Grosso do Sul [7,9,10]. Recently, Martins et al. [10] found, for the first time, domiciliary colonies of *T. williami* in an urban area of Barra do Garças, Mato Grosso, Brazil. Its original description is short and poorly detailed, highlighting the black spots on intersegmental sutures in the dorsal connexivum, similar to *Triatoma sordida* (Stål, 1859) and *Triatoma guasayana* Wygodzinsky and Abalos, 1949 [9,11,12]. According to Lent and Wygodzinsky [13], *T. williami* differs from these two species in the following characters: pronotum, scutellum, and legs coloration; head length; length of the anteocular region in relation to the post-ocular; eye size; and length and shape of the scutellum.

*Triatoma guazu* Lent and Wygodzinsky, 1979 was described based on a single female specimen, found in a human dwelling, in the municipality of Villarica, Guairá Department, Paraguay, located in the Humid Chaco ecoregion at ~1000 km from Goiás, Brazil. Male specimens were described only ~20 years later based on bugs collected in the municipality of Barra do Garças, Mato Grosso, Brazil, located 75 km apart from the type locality of *T. williami* in the Cerrado [7,13,14]. According to Lent and Wygodzinsky [13], *T. guazu* resembles *Triatoma oliveirai* (Neiva, Pinto and Lent, 1939), “This species resembles *T. oliveirai*, but differs from the latter by many characters, such as the short rostral setae, the larger eyes and ocelli, the unicolorous pronotum, the horizontal scutellar process, the no abbreviated hemelytra, and the quite different shape of the ninth urotergite”.

Noireau et al. [15] observed a great similarity between sympatric specimens of *T. williami* and *T. guazu*, collected in the male type locality of the latter species, when analyzing 18 isoenzyme loci and four measurements of each, the head and thorax structures through traditional morphometry. In regard to the genitalia of the species studied here, Lent et al. [14] described the male genitalia of *T. guazu* and Teves et al. [16] described the female genitalia of *T. williami*. The former authors illustrated their description with drawings, while the latter with Scanning Electron Microscopy images.

Their results were corroborated by subsequent molecular studies with the 12S [17] and 16S [17,18,19,20,21], and 18S and 28S subunits of the mitochondrial and cytoplasmatic ribosomal RNA (SSU rRNA), respectively [20,22], as well as the mitochondrial cytochrome oxidases I (COI) [19,20,21] and II (COII) [20] and Cytochrome B (CytB) [19,20]. All samples analyzed so far were from the municipality of Barra do Garças, Mato Grosso, Brazil, except the specimens used in two studies [18,20], which were from unknown locations. Chromosomal analysis indicated a similar number and the morphology of *T. guazu* and *T. williami* chromosomes [23], and fluorescent in situ hybridization (FISH) showed evidence that both species are concentrated in 45S rDNA clusters in an autosomal pair, and not in sexual chromosomes, such as observed in other *Triatoma* species such as *T. maculata* and *T. matogrossensis* [24]. Reis et al. [25], however, observed that the two species can be differentiated by the number of peripheral heteropyknotic filaments during spermiogenesis (a single filament in *T. williami* and two filaments in *T. guazu*). In the present study, we review the taxonomic *status* of *T. williami* and *T. guazu* through the analysis of geometric morphometry of head capsules, genetic divergence of publicly available rRNA and mtDNA sequences, and morphological analysis.

## 2. Materials and Methods

**Figure 1:**
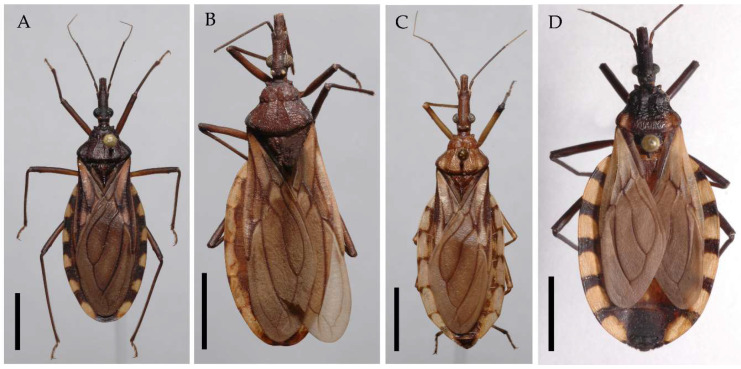
Species studied, dorsal view: (**A**) *Triatoma guazu*; (**B**) *Triatoma williami*; (**C**) *Triatoma matogrossensis*; (**D**) *Triatoma oliveirai*. Scale 5.0 mm.

**Figure 2:**
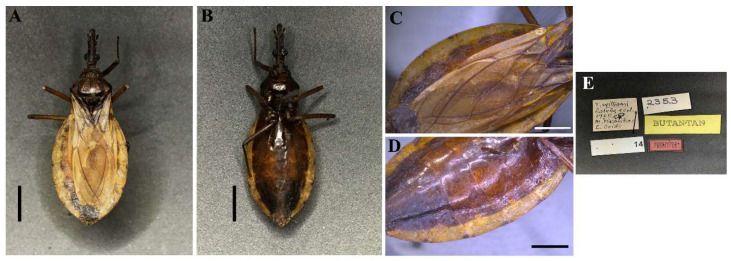
*Triatoma williami* (paratype, female, deposited in CEIB): (**A**) Dorsal view; (**B**) Ventral view; (**C**) Connexivum, dorsal view; (**D**) Connexivum, ventral view; (**E**) Labels. (**A**–**D**) Scale 5.0 mm.

**Figure 3:**
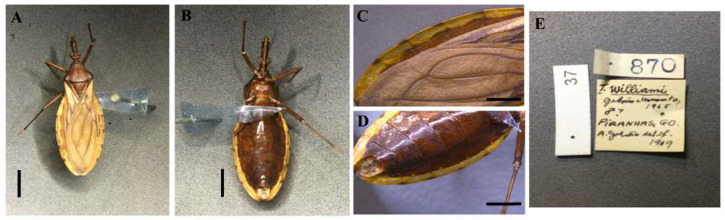
*Triatoma williami* (allotype, male, deposited in CEIB): (**A**) Dorsal view; (**B**) Ventral view; (**C**) Connexivum, dorsal view; (**D**) Connexivum, ventral view; (**E**) Labels. (**A**–**D**) Scale 5.0 mm.

**Figure 4:**
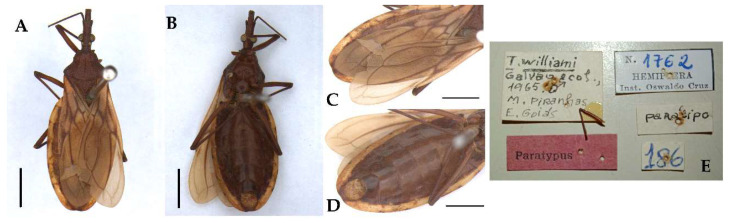
*Triatoma williami* (paratype, male, deposited in CTIOC): (**A**) Dorsal view; (**B**) Ventral view; (**C**) Connexivum, dorsal view; (**D**) Connexivum, ventral view; (**E**) Labels. (**A**–**D**) Scale 5.0 mm.

**Figure 5:**
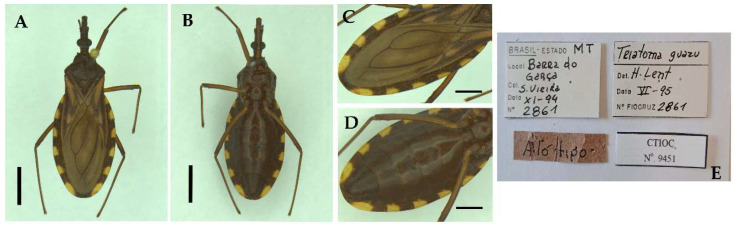
*Triatoma guazu* (allotype, male, deposited in CTIOC): (**A**) Dorsal view; (**B**) Ventral view; (**C**) Dorsal view of the connexivum; (**D**) Ventral view of abdomen, including; (**E**) Labels. (**A**–**D**) Scale 5.0 mm.

**Figure 6:**
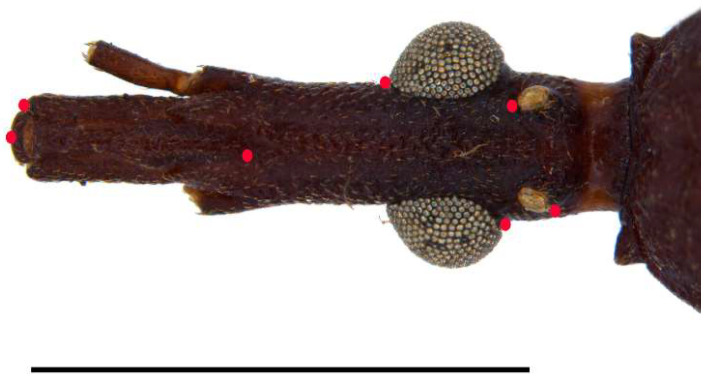
*Triatoma williami*, head, dorsal view of a showing landmarks (target spots) used in morphometric analysis. Scale 5.0 mm.

**Figure 7:**
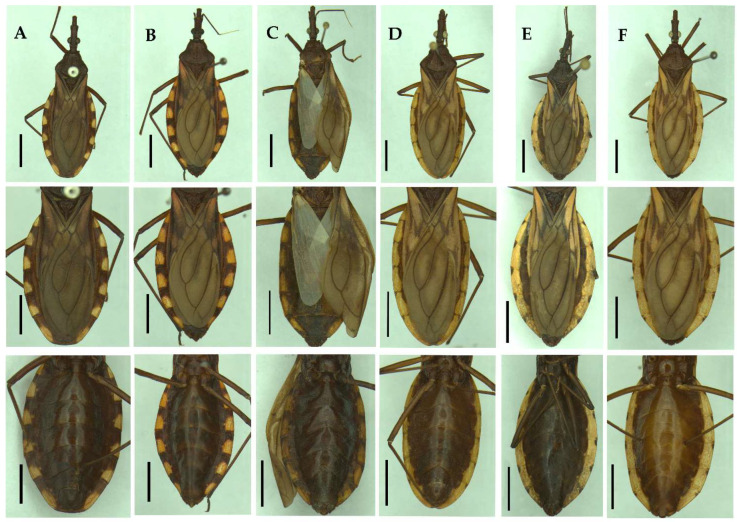
Variation of the connexivum spotting pattern. (**A**–**C**) *Triatoma guazu*, wide dark spots, in dorsal and ventral views; (**D**–**F**) *Triatoma williami*, narrow dark spots, in dorsal and ventral views. Scale 5.0 mm.

Morphological analysis. We examined 44 individuals (12 *Triatoma guazu* and 32 *T. williami*) including the type specimens, deposited in Coleção Entomológica do Instituto Butantan (CEIB), São Paulo, Brazil, and Coleção de Triatomíneos do Instituto Oswaldo Cruz (CTIOC), Fiocruz, Rio de Janeiro, Brazil (see examined material in Supplemental Data S1, Figure 1, Figure 2, Figure 3, Figure 4 and Figure 5). Photographs and morphological analysis were taken with a Leica DMC 2900 camera attached to a Leica M205C stereomicroscope (Figure 1, Figure 4, Figure 5, Figure 6 and Figure 7). Images were edited using Adobe Photoshop 7.0.1. General morphological terminology mainly follows original descriptions of the species [9,12] and previous studies on Triatominae (e.g., Lent and Wygodzinsky [13]).

Geometric morphometrics. The adult head capsule was analyzed from 12 specimens of *T. guazu* (from Mato Grosso State) and 20 of *T. williami* (from the States of Goiás, Mato Grosso and Mato Grosso do Sul), in addition to the outgroup consisting of 20 specimens of *T. matogrossensis* (from Mato Grosso State) and three specimens of *T. oliveirai* (from the Rio Grande do Sul State) (see examined material in Supplemental Data S1, Figure 1). The head was photographed using a digital camera Nikon coolpix 990, and the landmark coordinates were recorded with TpsDig version v. 2.05 (New York, United States) [26] to achieve a better definition of the head capsule conformation of the specimens [26] (Figure 6). Landmarks were superimposed to Generalized Procrustes Analysis in TPsRelw 1.53 [27,28,29,30]. This method allows the calculation of shape variables among taxa after alignment of the landmarks (to ensure homology). The multivariate differences were evaluated by the Lambda test of Wilks. The scores matrix was examined by Canonical Variate Analysis (CVA) to plot and observe the position of each specimen on the “Shape Discriminant Space”. The observed average distances were used to analyze the relationship among the species through the reconstruction of an UPGMA dendrogram. All analyses were executed in JMP 3.2.6 (SAS Institute, Cary, NC, USA).

Analysis of male genitalia. The dissections of a male genitalia of a specimen identified as *T. williami* were made by first removing the pygophore from the abdomen with a pair of forceps and then clearing it in 20% NaOH solution for 24 h. The dissected structures were studied and photographed in glycerol (see the label of the analyzed specimen at Data S1). The photographs were obtained using a digital camera (Sony DSC-W830, Budapest, Hungary). Images were edited using Adobe Photoshop CS6.

In relation to the male genitalia portions, the terminology used here follows mostly Lent and Wygodzinsky [13]. However, “vesica” as recognized by Lent and Wygodzinsky [13] and Lent et al. [14] has been considered to be absent in reduviids. The assumed equivalent structure in reduviids is a somewhat sclerotized appendage of the phallosoma or the endosoma [31], but not the homologous vesica that occurs in other heteropterans such as Pentatomomorpha [32]. Thus, this term is not used here for the median process of endosoma, which is named as such.

Scanning Electron Microscopy. The samples of *T. guazu* examined were obtained from colonies maintained by the Laboratório Nacional e Internacional de Referência em Taxonomia de Triatomíneos, Instituto Oswaldo Cruz, Rio de Janeiro, Brazil (see the label of the analyzed specimens at Data S1). The females were washed with detergent and subsequently metallized. Then, cross-sections were performed between abdominal tergites II and III. The genitals were dehydrated in an alcoholic series on silica and fixed in aluminum stubs. The genitalia were plated on the Rudolf Barth Plataforma de Microscopia Eletrônica de Varredura (IOC) and were photographed using the microscopes JEOL JSM-6390LV.

Molecular analysis. A total of 64 DNA sequences of *Triatoma brasiliensis* Neiva, 1911 [33], *T. guazu, Triatoma jatai* Gonçalves, Teves-Neves, Santos-Mallet, Carbajal-de-la-Fuente, and Lopes, 2013 [34], *T. matogrossensis* and *T. williami* were retrieved from GenBank [35] for 16S, COI, COII and CytB. *Triatoma oliveirai* is a rare species and does not have any DNA sequence publicly available. DNA sequence data were aligned separately for each marker using ClustalW [36] implemented in the MEGA-X [37]. MEGA-X was used then to identify the number of variable sites. A pairwise divergence calculation was performed in the MEGA-X program, using the bootstrap method of resampling with 500 replications and the Kimura-2-parameters as the substitution model [38]. Species comparisons were performed by calculating the difference between inter and intraspecific genetic distances. Here, we considered as ‘true’ species those which interspecific divergences were above intraspecific divergences observed for *Triatoma* species.

## 3. Results

In the present study, we highlight new diagnostic characters among these species, in addition to those mentioned by Lent and Wygodzinsky [13]: length of the anteocular region in relation to the postocular region; eye position in relation to the ventral surface of the head; length of the submedian carina of the pronotum; and absence of spongy fossa (see Supplemental Table S2). The morphological data did not reveal diagnostic characters to differentiate between *T. guazu* and *T. williami*, except for the connexivum spots pattern (see Supplemental Data S2). Based on the original description, both species in the dorsal view have spots on the intersegmental sutures of the connexivum, which are dark and wide in *T. guazu*, and dark and narrow in *T. williami*. However, the present analysis evidenced a continuous variation, within the populations of the two species, from narrow to wide dark spots (Figure 7).

**Figure 8:**
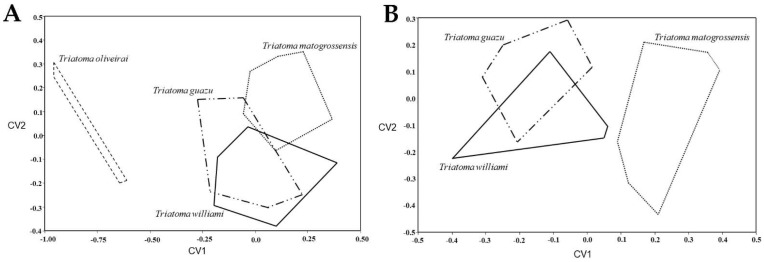
Factorial map built with the first two canonical vectors (CV) showing the head shape discrimination. (**A**) Between *Triatoma guazu*, *Triatoma williami*, *Triatoma matogrossensis*, and *Triatoma oliveirai*; (**B**) Without *T. oliveirai*.

The discriminant analysis of the head capsule shape showed that specimens originally designated as *T. guazu* are not distinct from *T. williami* specimens (Figure 8A,B).

**Figure 9:**
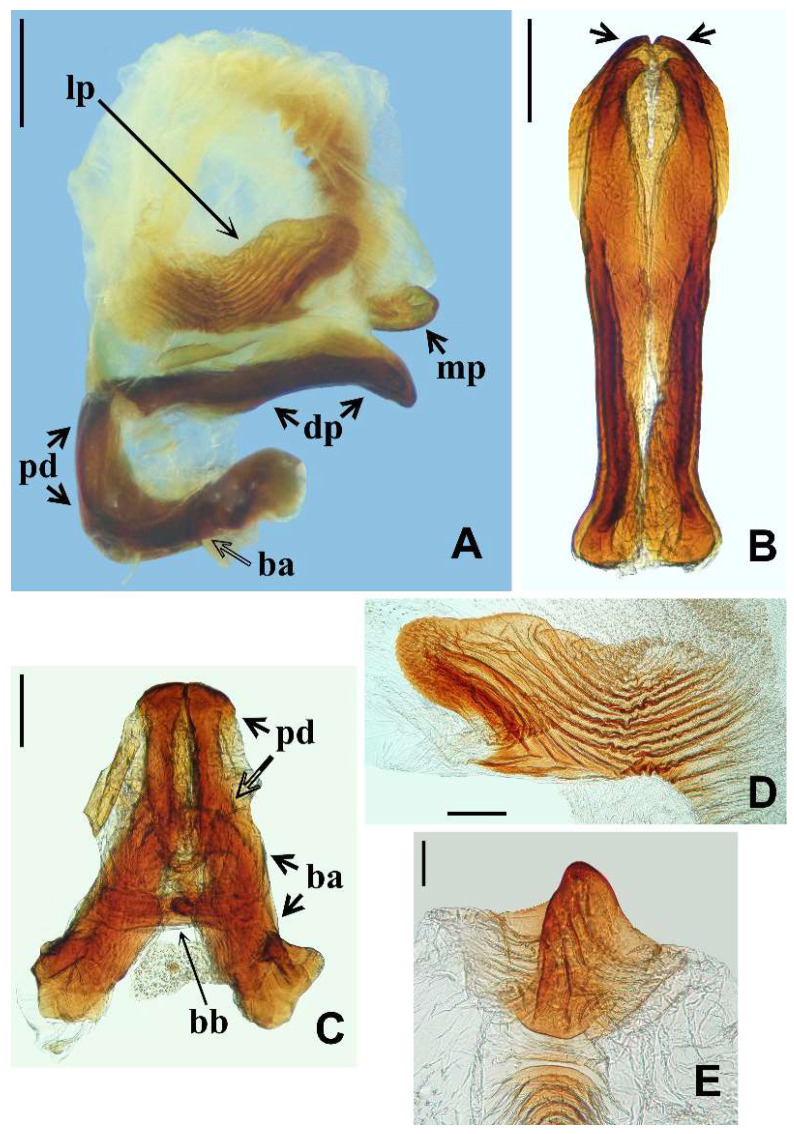
*Triatoma williami*, male genitalia. (**A**) Phallus, lateral view, scale bar 0.5 mm. (**B**,**C**) Dorsal view, scale bar 0.3 mm. (**B**) Struts and apical portion of phallothecal plate (pointed by arrows). (**C**) Articulatory apparatus and pedicel. (**D**) Lateral process of endosoma, lateral view, scale bar 0.2 mm. (**E**) Median process of endosoma, superior view, scale bar 0.1 mm. (ba, basal plate arm; bb, basal plate bridge; dp, dorsal phallothecal plate; lp, lateral process of endosoma; mp, median process of endosoma; pd, pedicel).

The male genitalia of a specimen identified as *T. williami* (Figure 9A–E) revealed the same characteristics as those described to *T. guazu* [14]. It is noteworthy that the mentioned uniformity was recorded in the structures which are usually more important to diagnosis or to distinguish species of *Triatoma*, such as the phallic portions (articulatory apparatus, pedicel, phallothecal plate, including the struts and the processes of the endosoma) (Figure 9A–E), which presented the same shape, dimensions, and peculiarities in *T. williami* as described in *T. guazu* by Lent et al. [14].

**Figure 10:**
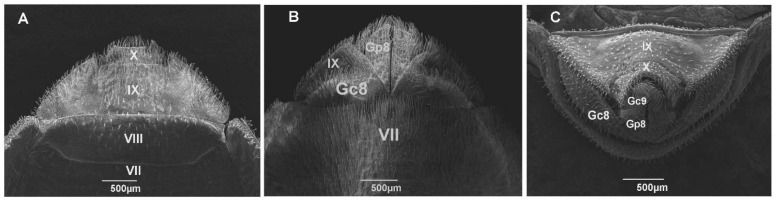
Female external examined by scanning electron microscopy of *Triatoma guazu*. (**A**) Dorsal view (VII and VIII, tergites; IX and X segments). (**B**) Ventral view (VII, sternite; IX, segment; Gc8, gonocoxite VIII; Gp8, gonapophysis VIII). (**C**) Posterior view: (Gc8, gonocoxite VIII; Gc9, gonocoxite IX; Gp8, gonapophysis VIII; IX and X, segments).

In the same way, the character set of the female genitalia of *T. guazu* did not reveal differences when compared to *T. williami*, genitalia described by Teves et al. [17] (Figure 10A–C). It was not possible to compare the genitalia in posterior view, because there is no description for *T. williami*. However, in posterior view, we observed the following characters for *T. guazu*: appendix not visible; gonocoxite VIII elongated and slightly wider; abdominal segments IX and X slightly downward and as wide as long; and tergite IX posterior margin clearly separated from tergite X.

A total of 56 sequences were selected and analyzed (Table 1): 16S gene fragments (20 sequences, 356 bp, 48 variable sites); COI (16 sequences, 201 bp, 52 variable sites); COII (eight sequences, 284 bp, and 79 variable sites); and CytB (12 sequences, 313 bp, and 91 variable sites). In general, those markers provide strong evidence that *T. guazu* and *T. williami* are the same species. Pairwise comparisons revealed that sequences of these two species diverge less than 2% for all markers analyzed (16S = 0.2%, COI = 1.5%, COII = 1.7%, CytB = 0.9%). These levels of divergence were below some intraspecific comparisons in *T. matogrossensis* (see Table 2, Table 3, Table 4 and Table 5; see Supplemental Table S2A–D) and *T. brasiliensis* (see Table 5; Table S2A). A similar pattern was found for COII and CytB pairwise divergences, which *T. guazu* and *T. williami* diverged 1.7% and 0.9%, respectively, and intraspecific divergences within *T. matogrossensis* reached up to 3.2% in COII sequences, and 1.2% in intraspecific divergences within *T. brasiliensis* CytB sequences.

## 4. Discussion

Lent and Wygodzinsky [13] considered *T. oliveirai* to be the closet taxon of *T. guazu*, because they have connexival spots in a similar pattern. In contrast, our morphological review confirms that *T. guazu* and *T. oliveirai* are not a closely related species.

In contrast, the low genetic divergence, lack of morphological diagnostic characters, and low morphometric distinction observed between *T. guazu* and *T. williami* corroborate previous studies which stated that they are very similar taxa [14,15,16,17,18,19,20,21,22]. Additionally, the variation in the spots pattern in the connexivum is compatible with phenotypic variations between populations of the same species, possibly a consequence of different ecological characteristics, not standing as a feature to consider them as a separate species [14,39].

The close phylogenetic relationship between *T. guazu* and *T. williami* had already been addressed in previous studies [15,19,20,21,22], being confirmed in phylogenetic reconstructions with high node support. Almeida et al. [40] reported low genetic divergence for species from southern Brazil, which may be the result of recent speciation events. Previous studies used 16S, COI, COII, and CytB, with fragments between 200 and 300 bp, to separate species from *Triatoma brasiliensis* [41], *Triatoma matogrossensis* [19] and *Triatoma rubrovaria* subcomplexes [40], highlighting the effectiveness of our approach.

Populations of *T. guazu* and *T. williami* occur in the same municipality of Barra do Garças and their biology remains unknown. These sympatric populations show chromatic variations, but chromatic differences provide weak evidence to support distinct species. *Triatoma infestans* var. *melanosoma* Lent, Jurberg, Galvão, and Carcavallo, 1994 [42] and *Triatoma infestans* sensu stricto (Klug, 1834) [43] have sympatric populations, exhibit different color patterns and are the same species [39,44]. Conversely, individuals from different populations of *Triatoma rubrovaria* (Blanchard, 1843) [45] are polychromatic [13,46,47]. Indeed, Dale et al. [47] observed 16 different chromatic patterns in the collar, pronotum, and connexivum of this species, questioning the validity of these variations as diagnostic characters to designate new species.

Despite the chromatic patterns, which seems to be a continuous variation, the distinct number of heteropyknotic filaments in spermatogenesis is the only character to our knowledge that differentiate *T. guazu* from *T. williami* [25,48]. Perhaps both phenotypes are present in the two species and more research involving a better sampling strategy with sympatric and allopatric populations could shed light on this subject. We also emphasize the importance of population genetics studies to identify the presence (or the lack of) barriers to the gene flow of specimens with different chromatic patterns. So far, morphological traits, traditional and geometric morphometry, isozyme and DNA sequencing data prove that *T. guazu* is a junior synonym of *T. williami*.

## 5. Conclusions

Finally, we emphasize the importance of population genetics studies to identify the presence (or the lack of) barriers to the gene flow of specimens with different chromatic patterns. So far, morphological traits, traditional and geometric morphometry, comparison of genitalia in SEM, isozyme and DNA sequencing data prove that *T. guazu* is a junior synonym of *T. williami*.

**Taxonomy**

Order Hemiptera

Suborder Heteroptera

Family Reduviidae

Subfamily Triatominae

Tribe Triatomini

Genus *Triatoma* Laporte, 1832

*Triatoma williami* Galvão, Souza, and Lima, 1965

*Triatoma guazu* Lent and Wygodzinsky, 1979, **syn. nov**.

## Figures and Tables

**Table 1:** Specimens examined, locality information (when available), ID (unique specimen identifier number), marker and GenBank accession numbers.

Species	ID	Geographic Origin	Markers			
			16S rRNA	COI	COII	Cytb
*Triatoma brasiliensis*	40A	Curaçá, BA, Brazil	KC248986	KC249319	-	KC249240
	40B	Curaçá, BA, Brazil	-	KC249320	-	-
			-		-	-
	172	Tauá, CE, Brazil	-	KC249318	-	-
	41	Sobral, CE, Brazil	KC248987	-	-	KC249241
	174	Tauá, CE, Brazil	KC248985	-	KC249413	KC249239
*Triatoma guazu*		-	-	-	-	-
		Barra do Garças, MT, Brazil	-	-	KC249440	-
		Barra do Garças, MT, Brazil	-	-	-	-
		Barra do Garças, MT, Brazil	-	-	-	-
	29	Barra do Garças, MT, Brazil	KC249013	-	-	-
		Barra do Garças, MT, Brazil	-	KC608984	-	KC608976
*Triatoma jatai*	03	Paranã, TO, Brazil	KT601153	KT601162	-	-
	05	Paranã, TO, Brazil	KT601154	KT601163	-	-
	16	Paranã, TO, Brazil	KT601155	KT601164	-	-
*Triatoma juazeirensis*	209	Uiabi, BA, Brazil	KC249026	KF826892	-	KC249263
	CTA207	Juazeiro, BA, Brazil	KF769453	-	-	-
*Triatoma matogrossensis*		-	AF324525	-	-	-
		-	AF324526	-	-	-
		-	-			-
		Rio Verde de Mato Grosso, MS, Brazil	-	KC608985	-	KC608972
		Rio Verde de Mato Grosso, MS, Brazil	-	-	-	KC608978
	32	Aquidauana, MS, Brazil	KC249039	-	-	KC249271
	33	Alegria, MT, Brazil	KC249040	-	KC249460	-
	191	-	-	KC249359	KC249456	KC249269
	192	São Gabriel D’oeste, MS, Brazil	KC249037	KC249360	KC249457	KC249270
*Triatoma melanica*	Haplotype G	-	-	KJ580492		-
	Haplotype H	-		KJ580493		
	Haplotype I	-		KJ580494		
	Haplotype J	-		KJ580495		
		Urandi, BA, Brazil	KF769454	-		
		-	KC249041	-	KC249461	
*Triatoma sherlocki*	80		KC249068	-	KC249378	
			EU489057			EU489058
*Triatoma williami*	04	Barra do Garças, MT, Brazil	KT601156	KT601165	-	-
	36	-	KC249089	-	KC249493	-
		Barra do Garças, MT, Brazil	-	-	-	-
		Barra do Garças, MT, Brazil	-			KC608981
		-	-	-	-	-

(-) Uninformative.

**Table 2:** Average divergence estimates obtained from pairwise comparisons among sequences of *T. guazu* and *T. williami* using the Kimura model 2- Parameter for 16S. Interspecific divergence between these two species are in boldface.

	1	2	3	4	5	6	7	8
1.*Triatoma guazu*	-							
2.*Triatoma williami*	0.002	**0.000**						
3.*Triatoma matogrossensis*	0.066 (0.065–0.068)	0.063 (0.062–0.065)	**0.005 (0.000–0.008)**					
4.*Triatoma jatai*	0.053	0.049	0.072 (0.071–0.074)	**0.000**				
5.*Triatoma brasiliensis*	0.054 (0.052–0.055)	0.054 (0.052–0.055)	0.084 (0.080–0.090)	0.064 (0.061–0.068)	**0.003 (0.028–0.005)**			
6.*Triatoma juazeirensis*	0.063 (0.062–0.065)	0.060 (0.059–0.062)	0.082 (0.077–0.090)	0.060 (0.058–0.061)	0.021 (0.019–0.025)	**0.002**		
7.*Triatoma melanica*	0.057 (0.055–0.059)	0.054 (0.052–0.055)	0.086 (0.083–0.090)	0.054 (0.052–0.055)	0.026 (0.022–0.028)	0.033 (0.031–0.034)	**0.002**	
8. *Triatoma sherlocki*	0.057 (0.056–0.059)	0.054 (0.053–0.056)	0.084 (0.080–0.089)	0.051 (0.049–0.052)	0.022 (0.019–0.024)	0.030 (0.028–0.031)	0.018 (0.017–0.019)	**0.000**

**Table 3:** Average divergence estimates obtained from pairwise comparisons among sequences of *T. guazu* and *T*. *williami* using the Kimura model 2- Parameter for COI. Interspecific divergence between these two species are in boldface.

	1	2	3	4	5	6	7
1. *Triatoma guazu*	-						
2. *Triatoma williami*	0.015 (0.000–0.015)	**0.015**					
3. *Triatoma matogrossensis*	0.123 (0.108–0.138)	0.126 (0.108–0.145)	**0.020 (0.000–0.030)**				
4. *Triatoma jatai*	0.090	0.088 (0.085–0.090)	0.147 (0.137–0.157)	**0.000**			
5. *Triatoma brasiliensis*	0.128 (0.125–0.131)	0.137 (0.125–0.151)	0.181 (0.161–0.201)	0.191 (0.187–0.194)	**0.005**		
6. *Triatoma melanica*	0.149	0.159 (0.149–0.169)	0.191 (0.181–0.202)	0.161	0.082 (0.079–0.085)	**0.000**	
7. *Triatoma juazeirensis*	0.126	0.126	0.133 (0.124–0.141)	0.167	0.049	0.076 (0.042–0.087)	-

**Table 4:** Average divergence estimates obtained from pairwise comparisons among sequences of *T. guazu* and *T. williami* using the Kimura model 2- Parameter for COII. Interspecific divergence between these two species are in boldface.

	1	2	3	4	5	6
1.*Triatoma guazu*	-					
2. *Triatoma williami*	0.017	-				
3.*Triatoma matogrossensis*	0.165 (0.162–0.167)	0.165 (0.162–0.171)	**0.022 (0.000–0.032)**			
4. *Triatoma brasiliensis*	0.156	0.143	0.168 (0.168–0.187)	-		
5. *Triatoma melanica*	0.166	0.156	0.245 (0.236–0.254)	0.152	-	
6. *Triatoma sherlocki*	0.164	0.155	0.206 (0.197–0.212)	0.116	0.091	-

**Table 5:** Average divergence estimates obtained from pairwise comparisons among sequences of *T. guazu* and *T. williami* using the Kimura model 2- Parameter for CytB. Interspecific divergence between these two species are in boldface.

	1	2	3	4	5	6
1. *Triatoma guazu*	-					
2. *Triatoma williami*	0.009	-				
3. *Triatoma matogrossensis*	0.153	0.140	**0.006 (0–0.006)**			
4. *Triatoma brasiliensis*	0.198	0.196	0.181 (0.178–0.187)	**0.012 (0–0.012)**		
5. *Triatoma sherlocki*	0.198	0.204	0.213	0.141 (0.141–0.149)	-	
6. *Triatoma juazeirensis*	0.174	0.178	0.148	0.093	0.012	-

## Data Availability

Publicly available datasets were analyzed in this study. This data can be found here: [http://www.ncbi.nlm.nih.gov/genbank/ (accessed on 20 June 2022) access codes are found in Table 1].

## Supplementary Materials

The following supporting information can be downloaded at: https://www.mdpi.com/article/10.3390/insects13070591/s1, Data S1: Examined material of the studied species; Data S2: *T. guazu* and *T. williami* character set; Table S1: Diagnostic character sets between *T. guazu* and *T. oliveirai*; Table S2: Estimative of pairwise divergences between sequences of the studied species for 16S, COI, COII and CytB.
