# Supplementary material for: Triatoma guazu Lent and Wygodzinsky Is a Junior Synonym of Triatoma williami Galvão, Souza and Lima
Source: Insects. 2022 Jun 28;13(7):591. doi: 10.3390/insects13070591 (PMC9318919; doi:10.3390/insects13070591)
Supplement: Supplementary file 1 [file insects-13-00591-s001.zip › insects-1732882-supplementary.pdf]

Supplementary material

Data S1. Examined material of the studied species

The boldface text represent specimens used for geometric morphometric analysis, red text represent specimen used for analyzed male genitalia and blue text for analyzed female genitalia. A slash (/), separates the lines, a double slash (//) different labels and a semicolon (;) separates the specimens. The material is listed as per the original specimen label.

Triatoma guazu

Type material. Triatoma guazu / det. H. Lent/ Data: VI-95/ Nº. FIOCRUZ 2861// BRASIL- ESTADO MT/ Local Barra do/ Garça/ Col. S.Vieira/ Data XI-94/ Nº2861// Alótipo // CTIOC / N° 9451 ♂.

Non-type material. T. guazu // 2617 // CTIOC / Nº. 6750 ♀; FIOCRUZ / Instituto Oswaldo Cruz / Barra do Garças – MT / 23/11/98 ♂ // Triatoma / guazu / Lent & Wygod. / R. Carcavallo det.1998 // 5514 // CTIOC / N°.6746; FIOCRUZ / Instituto Oswaldo Cruz / Barra do Garças / MT / 23/11/98 ♀ // Triatoma / guazu / Lent & Wygod. / R. Carcavallo det.1998 // 5512 // CTIOC / Nº. 6745; FIOCRUZ / Instituto Oswaldo Cruz / Barra do Garças / 23/11/98 ♂ // Triatoma / guazu / Lent & Wygod / R. Carcavallo det.1998 //5315 //CTIOC / Nº.6747; FIOCRUZ / Instituto Oswaldo Cruz / T. guazu/ Barra das Garças/ Mato Grosso/ Criado em lab/ 08/97 //5520// CTIOC / Nº.6787 ♂; FIOCRUZ / Instituto Oswaldo Cruz / T. guazu/ Barra dos Garças/ Mato Grosso/ Criado em lab/ 08/97 // 5519 // CTIOC / Nº.6751 ♂; FIOCRUZ / Instituto Oswaldo Cruz / T. guazu/ Barra das Garças/ Mato Grosso/ Criado em lab/ 08/97 // 5518// CTIOC / Nº.6748 ♀; Brasil – Mato / Grosso – Barra /do Garça/ Laborat. // Triatoma / guazu/ Lent & Wyg- /R. Carcavallo det.1997 //5524 // CTIOC / Nº.6755 ♀; Brasil – Mato / Grosso – Barra /do Garça/ Laborat // Triatoma guazu / Lent & Wygodz- /1979/ R. Carcavallo det.1997 //5521// CTIOC/ Nº.6752 ♂; Brasil – Mato / Grosso – Barra /do Garça/ Laborat. // Lent & Wygodz- /1979/ R. Carcavallo det.1997 //5523// CTIOC /Nº. 6754 ♀; Brasil – Mato / Grosso – Barra /do Garça/ Laborat. // Lent & Wygodz- /1979/ R. Carcavallo det.1997 //5522// CTIOC /Nº. 6753 ♂; FIOCRUZ / Instituto Oswaldo Cruz / Barra do Garças / MT / 13/05/21 ♀ // Triatoma / guazu / Lent & Wygod. / JP. Correia det.2021 // CTIOC / Nº. 14000; FIOCRUZ / Instituto Oswaldo Cruz / Barra do Garças / MT / 13/05/21 ♀ // Triatoma / guazu / Lent & Wygod. / JP. Correia det.2021 // CTIOC / Nº. 14001; FIOCRUZ / Instituto Oswaldo Cruz / Barra do Garças / MT / 13/05/21 ♀ // Triatoma / guazu / Lent & Wygod. / JP. Correia det.2021 // CTIOC / Nº. 14002.

Triatoma matogrossensis

Non-type material. Coleção/ Rodolfo Carcavallo// 3884 //CTIOC/ Nº. 4605 ♀; BRASIL/ Mato Grosso/ LAB/ IV-83// Coleção/ Rodolfo Carcavallo// 3880// CTIOC/ Nº. 4606 ♀; BRASIL/ Mato Grosso/Lab./ V.83// Coleção/ Rodolfo Carcavallo//3881//CTIOC/ Nº 4607 ♂; Triatoma/ matogrossensis/ Leite y Barbosa/ R. Carcavallo det. 1987// BRASIL/ Mato Grosso/ LAB/ IV-83// Coleção/ Rodolfo Carcavallo// CTIOC/ Nº. 4608 ♀; Triatoma **mato-/grossensis/ Leite y**

Barbosa/ R. Carcavallo det. 1984// BRASIL/ Mato Grosso/ laboratório/ IV-84// 3883// Coleção/ Rodolfo Carcavallo// CTIOC/ Nº. 4609 ♀; BRASIL/ Mato Grosso/ laboratório/ IV-84// 3893// Coleção/ Rodolfo Carcavallo// CTIOC/ Nº. 4610 ♂; BRASIL/ Mato Grosso/ laboratório/ IV-84// 3890// Coleção/ Rodolfo Carcavallo// CTIOC/ Nº. 4611 ♀; Br. Mato Grosso/ Lab IILP/ IV.84//3897// Coleção/ Rodolfo Carcavallo/ /CTIOC/ Nº. 4613 ♂; Coleção/ Rodolfo Carcavallo// 3892// CTIOC/ Nº. 4614 ♀; Coleção/ Rodolfo Carcavallo// 3888// CTIOC/ Nº. 4616 ♂; Coleção/ Rodolfo Carcavallo// 3854// CTIOC/ Nº. 4621 ♂; Coleção/ Rodolfo Carcavallo// 3896// CTIOC/ Nº. 4622 ♂; BRASIL/ Mato Grosso/ LAB/ IV-84// Coleção/ Rodolfo Carcavallo// 3902// CTIOC/ Nº. 4626 ♀; BRASIL/ Mato Grosso/ LAB/ V-83// Coleção/ Rodolfo Carcavallo// 3903// CTIOC/ Nº. 4627 ♀; Coleção/ Rodolfo Carcavallo// 3905// CTIOC/ Nº. 4629 ♀; Coleção/ Rodolfo Carcavallo// 3906// CTIOC/ Nº. 4630 ♀; BRASIL/ Mato Grosso/ LAB/ IV-84// Coleção/ Rodolfo Carcavallo// 3908// CTIOC/ Nº. 4643 ♀; Coleção/ Rodolfo Carcavallo// 3909// CTIOC/ Nº. 4634 ♂; Triatoma mato-/ grossensis/ H. LENT det./ Miranda M. Grosso/ 7-79 domic./ J. V. Alves// CTIOC/ Nº. 4636 ♀; Triatoma mato-/ grossensis/ H. LENT det./ Miranda M. Grosso/ 7-79 domic./ J. V. Alves// CTIOC/ Nº. 4637 ♀.

Triatoma oliveirai

Type material. Porto Alegre// Holotypus//HOLO /TYPUS //34.// Eutriatoma oliveirai/ Neiva, Pinto+Lent, 1939/ Holotypo ♀/ H. LENT DET. // Nº1042 / HEMIPTERA/ Inst. Oswaldo Cruz// CTIOC/ Nº. 8973// Eutriatoma oliveirai/ Holótipo/ Nº1048.

Non-type material. exemp 1 // Nº1049 / HEMIPTERA/ Inst. Oswaldo Cruz // T. oliverai // CTIOC/ Nº. 12087 ♀; Triatoma / oliveirai / H. LENT det. // SAPUCAIA DO SUL- RS / MORRO SAPUCAIA/ 19-X-86/ BARCELLOS, A. // 1999 // T. oliveirai ♀

Triatoma williami

Type material. T. williami/ Galvão e col./ 1965 ♀/ M. Piranhas/ E. Goiás// 2353// BUTANTAN// 14// Paratypus; T. williami/ Galvão e col./ 1965 ♂/ M. Piranhas/ E. Goiás// 186// BUTANTAN// Paratypus// parátipo// Nº 1762/ HEMIPTERA/ Inst. Oswaldo Cruz; **T. williami/ Galvão e col./ 1965 ♂?/ Piranhas, Goiás/ A. Galvão del. 1969// 870// 37/ Alótipo ♂.**

Non-type material. T. williami??/ Det. J. Jurberg/ Data 7. 2006/ Nº FIOCRUZ// Barra do Garça, MT/ Serra do Roncador/ Col. Vanda Cunha/ 9. 2004 // CTIOC/ Nº 11063 ♀; T. williami/ Det. J. Jurberg/ Data 7. 2006/ Nº FIOCRUZ// Barra do Garça, MT/ Serra do Roncador/ Col. Vanda Cunha/ 9. 2004 // CTIOC/ Nº 11065 ♀; **T. williami??/ Det. J. Jurberg/ Data 7. 2006/ Nº FIOCRUZ// Barra do Garça, MT/ Serra do Roncador/ Col. Vanda Cunha/ 9. 2004 // CTIOC/ Nº 11067 ♂; T. williami??/ Det. J. Jurberg/ Data 7. 2006/ Nº FIOCRUZ// Barra do Garça, MT/ Serra do Roncador/ Col. Vanda Cunha/ 9. 2004 – criad. lab // CTIOC/ Nº 11068 ♀; T. williami Galvão,/ Souza & Lima, 1965/ Det. Bulhões, D. M/ Data 25.03.99/ Nº FIOCRUZ// BRASIL – ESTADO MT/ Local P. N. de Serra/ Azul B. do Garças/ Col. Bulhões, D. M/ Data 25.03.99/**

Nº 2926// CTIOC/ Nº 11066 ♀; T. williami Galvão,/ Souza & Lima, 1965/ Det. Bulhões, D. M/ Data 25.03.99/ Nº FIOCRUZ// BRASIL – ESTADO MT/ Local P. N. de Serra/ Azul B. do Garças/ Col. Bulhões, D. M/ Data 25.03.99/ Nº 1925// CTIOC/ Nº 11069 ♂; T. williami/ crist. Nº 4// CTIOC/ Nº 11083 ♂; **T. williami Galvão,/ Souza & Lima, 1965/ criado em lab./ abril 99// BRASIL – ESTADO GO/ Local Fazenda Nova/ Col. Janizete Garcia/ Data 07.95/ I. O. Cruz 5532// T. williami Galvão,/ Souza & Lima, 1965/ Det./ Data 14.04.99/ Nº FIOCRUZ 5532// CTIOC/ Nº 11021 ♀;** T. williami/ Det. Jóse Jurberg/ Data III. 2010/ Nº FIOCRUZ 3364// FIOCRUZ Instituto Oswaldo Cruz/ MT. Serra do/ Roncador – Barra/ do Garça- XV.2007/ Col Vanda Cunha/ Criado no insetario// CTIOC/ Nº 5592 ♀; T. williami/ Det. Jóse Jurberg/ Data III. 2010/ Nº FIOCRUZ 3365/ FIOCRUZ Instituto Oswaldo Cruz/ MT. Serra do/ Roncador – Barra/ do Garça- XV.2007/ Col Vanda Cunha/ Criado no insetario// CTIOC/ Nº 5593 ♂; T. williami/ Det. Jóse Jurberg/ Data III. 2010/ Nº FIOCRUZ 3363// FIOCRUZ Instituto Oswaldo Cruz/ MT. Serra do/ Roncador – Barra/ do Garça- XV.2007/ Col Vanda Cunha/ Criado no insetario// Cop. Herman Lent nº 3363// CTIOC/ Nº 5591 ♀; **T. williami Galvão,/ Souza & Lima, 1965/ Det. Bulhões, D. M/ Data 25.03.99/ Nº FIOCRUZ// BRASIL – ESTADO MT/ Local P. N. de Serra/ Azul B. do Garças/ Col. Bulhões, D. M/ Data 25.03.99/ Nº 5604// CTIOC/ Nº 10225 ♀;** T. williami Galvão,/ Souza & Lima, 1965/ Det. Bulhões, D. M/ Data 25.03.99/ Nº FIOCRUZ// BRASIL – ESTADO MT/ Local P. N. de Serra/ Azul B. do Garças/ Col. Bulhões, D. M/ Data 25.03.99/ Nº 5600// T. williami ♀; **T. williami Galvão,/ Souza & Lima, 1965/ Det. Bulhões, D. M/ Data 25.03.99/ Nº FIOCRUZ// BRASIL – ESTADO MT/ Local P. N. de Serra/ Azul B. do Garças/ Col. Bulhões, D. M/ Data 25.03.99/ Nº 5605// CTIOC/ Nº 110227 ♂; Triatoma williami ♀// 174// ♀// CTIOC/ Nº 11084; FIOCRUZ Instituto Oswaldo Cruz// T. williami/ criado em lab. 10.99// CTIOC/ Nº 11079 ♀;** T. williami/ Galvão e col.,/ 1965 ♀/ Aquidanana/ MG-Brasil/ capt. Sebastião/ A. Jonza-Dom./ B. Galvão det.// CTIOC/ Nº 11085; **BRASIL – ESTADO GO/ Local Faz. Nova/ col Donizete G. Silva/ Data VII/95/ Nº// 5448// CTIOC/ Nº 110211♀; BRASIL – Goiás/ Local M. de Sta/ Rita/ Data Set. 99/ I. O. Cruz.// CTIOC/ Nº 11080 ♀;** **T. williami Galvão,/ Souza & Lima, 1965/ Det. Bulhões, D. M/ Data 25.03.99/ Nº FIOCRUZ// BRASIL – ESTADO MT/ Local P. N. de Serra/ Azul B. do Garças/ Col. Bulhões, D. M/ Data 25.03.99/ Nº 2994// CTIOC/ Nº 11062 ♂;** T. williami Galvão,/ Souza & Lima, 1965/ Det. Bulhões, D. M/ Data 25.03.99/ Nº FIOCRUZ// BRASIL – ESTADO MT/ Local P. N. de Serra/ Azul B. do Garças/ Col. Bulhões, D. M/ Data 25.03.99/ Nº 5603// CTIOC/ Nº 11085 ♂; **T. williami Galvão,/ Souza & Lima, 1965/ Det. Bulhões, D. M/ Data 25.03.99/ Nº FIOCRUZ// BRASIL – ESTADO MT/ Local P. N. de Serra/ Azul B. do Garças/ Col. Bulhões, D. M/ Data 25.03.99/ Nº 5603// CTIOC/ Nº 10224 ♂; T. williami??/ Det. J. Jurberg/ Data 2006// Barra do Garça, MT/ Serra do Roncador/ Col. Vanda Cunha/ 9. 2004 // CTIOC/ Nº 11070 ♂;** T. williami??/ Det. J. Jurberg/ Data 2006// Barra do Garça, MT/ Serra do Roncador/ Col. Vanda Cunha/ 9. 2004 // CTIOC/ Nº 11072 ♀; **Triatoma williami// Barra do Garças-MT/ Col. Mirian F. Martins/ 02.X.19/ Det. Cleber Galção// Reared in LNIRTT/ Rio de Janeiro-RJ/ Inst. Oswaldo Cruz// CTIOC/ Nº 13007 ♀; Triatoma williami// Barra do Garças-MT/ Col. Mirian F. Martins/ 02.X.19/ Det. Cleber Galção// Reared in LNIRTT/ Rio de Janeiro-RJ/ Inst. Oswaldo Cruz// CTIOC/ Nº 13009 ♀; Triatoma williami// Barra do Garças-MT/ Col. Mirian F. Martins/ 02.X.19/ Det. Cleber Galção// Reared in LNIRTT/ Rio de Janeiro-RJ/ Inst. Oswaldo Cruz// CTIOC/ Nº 13008** ♂; **Triatoma williami// Barra do Garças-MT/ Col. Mirian F. Martins/ 02.X.19/ Det. Cleber Galção// Reared in LNIRTT/ Rio de Janeiro-RJ/ Inst. Oswaldo Cruz// CTIOC/ Nº 13006 ♀; Triatoma williami// Barra do Garças-MT/ Col. Mirian**

F. Martins/ 02.X.19/ Det. Cleber Galção// Reared in LNIRTT/ Rio de Janeiro-RJ/ Inst. Oswaldo Cruz// CTIOC/ Nº 13005 ♀.

Table S1. Diagnostic character sets between *T. guazu* and *T. oliveirai.*

Diagnostic character	*T. guazu*	*T. oliveirai*
Length of the anteocular region	Four times longer than the postocular	Three times longer than the postocular
Eye position in relation to the head, in lateral view	Slightly exceeding the level of the ventral and dorsal surface	Not exceeding the level of the ventral surface and far from the dorsal
Length of the submedian carina of the pronotum	Ending near or reaching the distal margin of the posterior lobe	Limited to the submedian region of the posterior lobe
Spongy fossa	Absent in females	Absent in males (unanalyzed female)

Data S2. *T. guazu and T. williami* character set

Character list: Characters analyzed for *T. guazu* and *T. williami.* Character 9 (red status) indicates the only difference between species.

Coating

1- Body surface, bristles: Short and sparse or not very apparent

2- Labia, segments III and IV (second and third segments visible, respectively), bristles, in lateral view: Short

Coloration

3- Head, spots, in dorsal view: Absent

4- Neck, spots, in dorsal view: Present

5- Neck, 1+1 spots, in dorsal view: White dotted

6- Pronotum, collar, spots, in dorsal view: Absent

7- 9- Pronotum, posterior lobe, predominant color, in dorsal view: Dark

8- Connexivum, dorsal plates, spots: Present

9- Connexivum, dorsal plates, pattern of spots: for *T. guazu*, dark and wide over the intersegmental sutures; *T. williami,* dark and narrow over the intersegmental sutures

10- Abdomen, color pattern, in ventral view: Simple

11- Femurs, spots: Absent

12- Tibias, spots: Absent

13- Tibias, spots: Absent

Integument texture

14- Head, synthlipsis, in dorsal view: Strongly rough

15- Head, region between tubercle and eye, in side view: Rough

16- Pronotum, posterior lobe, in dorsal view: Rough

17- Thorax, propleura and metapleura, in lateral view: Rough

18- Abdomen, sternites: Striated

Head

19- Head, shape, in dorsal view: Subcylindrical

20- Head, length in relation to width at eye level, in dorsal view: Twice or longer than wide

21- Head, length in relation to the length of the pronotum, in dorsal view: As long as or longer

22- Anteocular region, length in relation to the post-ocular, in dorsal view: More than four to five times longer

23- Postocular region, shape of the margins, in dorsal view: Subrectilinear or weakly convex

24- Maxillary plate, length in relation to the apical margin of the postclypeus, in dorsal view: Greater

25- Mandibular plate, tooth-like, in dorsal view: Discreet

26- Mandibular plate, length in relation to the maxillary plate, dorsal view: At least 1/3 to 1/2

27- Labia, third segment visible, length compared to second: Shorter

28- Labia, first visible segment, apex in relation to the antenniferous tubercle, in lateral view: Not reaching the level of the anterior margin

29- Labia, second visible segment, apex in relation to the eye, in lateral view: Exceeding or reaching the level of the posterior margin

30- Lip, third segment visible, shape of the apex, in ventral view: Tapered

31- Eye, in relation to the ventral surface of the head, in lateral view: Reaching or surpassing it

32- Eye, length in relation to the total length of the head, dorsal view: Less than or equal to 1/4

33- Eye, width in relation to the synthlipsis, in dorsal view: Smaller

34- Postocular region, approximation between the eyes, dorsal view: Proximal

35- Antenniferous tubercles, location, in dorsal view: Median, between the apex of the head and the eyes

36- First antennal segment, position of the apex in relation to the level of the clypeus, in dorsal view: Not reaching it

37- Third antennal segment, length compared to second: Shorter

Pronotum

38- Pronotum, submedian carina, length: Ending near or reaching distal margin of posterior lobe

39- Pronotum, lateral carina, in dorsal view: Absent

40- Pronotum, anterolateral angles, length and width proportion: Wider than long;

41- Pronotum, anterolateral angles, direction in relation to the longitudinal cephalic axis, in dorsal view: Divergent

43- Pronotum, anterior lobe, basal disc tubercles, in dorsal view: Absent or obsolete

44- Pronotum, anterior lobe, lateral tubercles, in dorsal view: Absent or obsolete

45- Pronotum, posterior lobe, towards the humeral angles, in lateral view: Not elevated;

46- Scutellum, central depression, in dorsal view: Well developed

47- Scutellum, posterior process, length in relation to the main body of the scutellum, in dorsal view: Shorter

48- Scutellum, posterior process, shape, in dorsal view: Conical

49- Scutellum, posterior process, form, in lateral view: Straight, slightly elevated;

50- Scutellum, posterior process, shape of the apex, in lateral view: Rounded

51- Prosternum, prosternal groove, shape: Short and wide

52- Prosternum, prosternal sulcus, lateral margins: Straight, parallel, narrowing in the apical third

53- Wing, hemelytra, length, in dorsal view: Reaching or slightly surpassing the apex of the seventh urotergite

54- Wing, small branch connecting basal portion R+M to S: Present

Abdomen

55- Abdomen, width in relation to total body length: Less than half

57- Abdomen, suture of the urosternite plate in relation to the dorsal connective plate, ventral view: Distal

58- Connective, ventral plates: Visible

59- Connective, ventral plates: Perceptible, similar in width to that displayed dorsally

Legs

60- Anterior and median femur, denticles: Present

62- Tibia, fossulae spongy: Present only in males

Table S2. A–D. Estimative of pairwise divergences between sequences of the studied species for 16S, COI, COII and CytB.

A. 16S

	1	2	3	4	5	6	7	8	9	10	11	12	13	14	15	16	17	18
1.*Triatoma guazu*_29																		
2.*Triatoma williami*_04	0,003																	
3.*Triatoma williami*_36	0,003	0,000																
4.*Triatoma matogrossensis*_192	0,065	0,062	0,062															
5.*Triatoma matogrossensis*_33	0,065	0,062	0,062	0,000														
6.*Triatoma matogrossensis*_32	0,069	0,065	0,065	0,008	0,008													
7.*Triatoma jatai*_03	0,053	0,050	0,050	0,071	0,071	0,075												
8.*Triatoma jatai*_05	0,053	0,050	0,050	0,071	0,071	0,075	0,000											
9.*Triatoma jatai*_16	0,053	0,050	0,050	0,071	0,071	0,075	0,000	0,000										
10.*Triatoma brasiliensis*_40	0,053	0,053	0,053	0,084	0,084	0,087	0,065	0,065	0,065									
11.*Triatoma brasiliensis*_41	0,056	0,056	0,056	0,087	0,087	0,090	0,068	0,068	0,068	0,003								

12.*Triatoma brasiliensis*_174	0,0560,0560,0560,0810,0810,0840,0620,0620,0620,0030,006	
13.*Triatoma juazeirensis*_2017	0,0650,0620,0620,0810,0810,0900,0620,0620,0620,0230,0260,020	
14.*Triatoma juazeirensis*_209	0,0620,0590,0590,0770,0770,0870,0590,0590,0590,0200,0230,0170,003	
15.*Triatoma melanica*_CTA206	0,0590,0560,0560,0870,0870,0900,0560,0560,0560,0260,0290,0290,0320,035	
16.*Triatoma_melanica*	0,0560,0530,0530,0840,0840,0870,0530,0530,0530,0230,0260,0260,0350,0320,003	
17.*Triatoma sherlocki*	0,0600,0560,0560,0860,0860,0890,0530,0530,0530,0210,0240,0240,0300,0300,0180,018	
18. *Triatoma sherlocki* 80	0,0560,0530,0530,0810,0810,0840,0500,0500,0500,0200,0230,0230,0320,0290,0200,0170,000	-

B. COI

	1	2	3	4	5	6	7	8	9	10	11	12	13	14	15	16	17
1.*Triatoma guazu*																	
2.*Triatoma williami*_4	0,015																
3.*Triatoma williami*_5	0,000	0,015															
4.*Triatoma* matogrossensis_192	0,108	0,114	0,108														
5.*Triatoma_matogrossensis*	0,139	0,145	0,139	0,031													
6.*Triatoma matogrossensis*_31	0,139	0,145	0,139	0,031	0,000												
7.*Triatoma matogrossensis*_191	0,108	0,114	0,108	0,000	0,031	0,031											
8.*Triatoma jatai*_16	0,091	0,085	0,091	0,138	0,157	0,157	0,138										
9.*Triatoma jatai*_05	0,091	0,085	0,091	0,138	0,157	0,157	0,138	0,000									
10.*Triatoma jatai*_03	0,091	0,085	0,091	0,138	0,157	0,157	0,138	0,000	0,000								
11.*Triatoma brasiliensis*_40	0,125	0,144	0,125	0,161	0,194	0,194	0,161	0,188	0,188	0,188							
12.*Triatoma brasiliensis*_172	0,131	0,150	0,131	0,168	0,201	0,201	0,168	0,194	0,194	0,194	0,005						
13.*Triatoma melanica*_J	0,150	0,169	0,150	0,181	0,202	0,202	0,181	0,162	0,162	0,162	0,085	0,080					
14.*Triatoma melanica*_I	0,150	0,169	0,150	0,181	0,202	0,202	0,181	0,162	0,162	0,162	0,085	0,080	0,000				
15.*Triatoma melanica*_H	0,150	0,169	0,150	0,181	0,202	0,202	0,181	0,162	0,162	0,162	0,085	0,080	0,000	0,000			
16.*Triatoma melanica*_G	0,150	0,169	0,150	0,181	0,202	0,202	0,181	0,162	0,162	0,162	0,085	0,080	0,000	0,000	0,000		
17.*Triatoma juazeirensis*	0,127	0,127	0,127	0,125	0,141	0,141	0,125	0,168	0,168	0,168	0,050	0,050	0,087	0,087	0,087	0,042	-

C. COII

	1	2	3	4	5	6	7	8	9
1.*Triatoma guazu*_29									
2.*Triatoma williami*_36	0,018								
3.*Triatoma matogrossensis*_192	0,167	0,162							
4.*Triatoma matogrossensis*_33	0,162	0,167	0,033						
5.*Triatoma matogrossensis*_31	0,166	0,171	0,033	0,000					
6.*Triatoma matogrossensis*_191	0,167	0,162	0,000	0,033	0,033				
7.*Triatoma brasiliensis*_174	0,157	0,143	0,169	0,183	0,188	0,169			
8.*Triatoma melanica*	0,167	0,156	0,236	0,254	0,254	0,236	0,152		
9.*Triatoma sherlocki*_80	0,165	0,156	0,197	0,213	0,218	0,197	0,117	0,092	-

D. CytB

	1	2	3	4	5	6	7	8	9	10	11	12
1.*Triatoma guazu*												
2.*Triatoma williami*	0,010											

3.*Triatoma matogrossensis*_32	0,153	0,140										
4.*Triatoma matogrossensis*_192	0,153	0,140	0,006									
5.*Triatoma matogrossensis*_191	0,153	0,140	0,006	0,000								
6.*Triatoma matogrossensis*	0,153	0,140	0,006	0,006	0,006							
7.*Triatoma matogrossensis*_kc608978	0,153	0,140	0,006	0,006	0,006	0,000						
8.*Triatoma brasiliensis*_174	0,192	0,196	0,187	0,187	0,187	0,187	0,187					
9.*Triatoma brasiliensis*_40	0,192	0,196	0,178	0,178	0,178	0,178	0,178	0,013				
10.*Triatoma brasiliensis*_41	0,192	0,196	0,178	0,178	0,178	0,178	0,178	0,013	0,000			
11.*Triatoma sherlocki*	0,192	0,204	0,213	0,213	0,213	0,213	0,213	0,150	0,141	0,141		
12.*Triatoma juazeirensis*_209	0,174	0,178	0,149	0,149	0,149	0,149	0,149	0,094	0,094	0,094	0,129	-
